# Prevalence and Factors Influencing Antiretroviral Therapy (ART) Nonadherence in Men During Different Stages of the Life Course: A Case Study in Greater Gaborone, Botswana

**DOI:** 10.1155/arat/5711642

**Published:** 2025-12-11

**Authors:** Matlhogonolo Kelepile, Sue C. Grady

**Affiliations:** ^1^ Department of Environmental Science, Faculty of Science, University of Botswana, Gaborone, Botswana, ub.bw; ^2^ Department of Geography, Environment, and Spatial Sciences, College of Social Sciences, Michigan State University, East Lansing, Michigan, USA, msu.edu

**Keywords:** AIDS, antiretroviral adherence, Botswana, Greater Gaborone, HIV, life-course, Treat All program

## Abstract

**Background:**

Adult men living with HIV are less likely than women to adhere to antiretroviral therapy (ART). This highlights the need to understand factors driving nonadherence and how adherence behaviors and barriers change with age. However, existing HIV surveillance data often collapse ages into broad groups, limiting life‐course analysis. This study addresses this gap by examining the time from HIV diagnosis to ART initiation and subsequent adherence barriers among men, using women as a comparison group, in Greater Gaborone, Botswana. To explore these dynamics, we employed a cross‐sectional survey design tailored to capture age‐specific patterns of ART initiation and adherence.

**Methods:**

A cross‐sectional survey was conducted with a stratified random sample of 239 men and 428 women attending 21 HIV treatment clinics. Semistructured questionnaires captured life‐course characteristics, including the stage of HIV disease at ART initiation and individual‐level factors affecting adherence.

**Results:**

The results showed that men (*n* = 239) initiated ART medication during Stage 1 (*n* = 165, 61.9%), early Stage 2 (*n* = 46, 19.2%), late Stage 2 (*n* = 17, 7.1%), and Stage 3 (*n* = 11, 4.6%). Most men (69.1%) were diagnosed in Stage 1 of HIV disease compared to early Stage 2 (19.2%), late Stage 2 (7.1%), and Stage 3 (4.6%). Majority of the men (*n* = 63, 38.2%) diagnosed in Stage 1 were aged 40–49 years, followed by those aged 30–39 years (*n* = 38, 23.0%). ART nonadherence rates were 46.0% for men and 29.1% for women. Men who were more likely to be ART nonadherent (compared to women) were aged 30–39 years (OR = 2.05, 1.06–3.56), 40–49 years (OR = 1.91, 1.06–3.45), and 50+ years (OR = 3.95, 1.73–8.97). Other risk factors for ART nonadherence were recently sick, comorbidities, and taking other medications.

**Conclusion:**

Despite free ART and medical care in Botswana, men face barriers related to comorbidities, highlighting the need for targeted gender‐specific interventions to improve ART adherence.

## 1. Introduction

### 1.1. Definition and Importance of Antiretroviral Therapy (ART) Adherence

The World Health Organization (WHO) defines ART adherence as the proportion of individuals consistently taking their antiretroviral (ARV) medications as prescribed [1]. Monitoring ART adherence is challenging without longitudinal studies. As a result, researchers rely on cross‐sectional and indirect measures such as self‐reporting, pill counts, pharmacy records, and biomonitoring [2–5]. ART adherence rates of at least 95% are critical to prevent treatment failure, viral mutation, and HIV transmission [6–8]. On a population level, we improved health translates to better livelihoods and economic growth in HIV‐affected regions. The scale‐up of ART across Africa has increased life expectancy (from 56 years in 2010 to 61 years in 2023) [9]. Among individuals living with HIV, including older adults, life expectancy has reached 70 years in South Africa [10, 11] and over 58 years in Eswatini [12].

### 1.2. Trends and Progress in HIV Care

The Joint United Nations Programme on HIV/AIDS (UNAIDS) [13] reports major progress through the “Treat All” program, which promotes ART initiation for all individuals diagnosed with HIV [13]. However, as of 2019, 12.6 million people (33.1%) of those living with HIV remained untreated, resulting in an estimated 260,000 AIDS‐related deaths largely due to delayed ART initiation [14]. Studies in sub‐Saharan Africa, including Cameroon [15] and Uganda [16, 17], have shown substantially higher mortality among those who delay treatment, particularly within the first year of ART. These findings highlight the ongoing need to address barriers to early diagnosis and timely treatment [18, 19].

### 1.3. Biological Implications on ART Nonadherence

Timely ART initiation after HIV diagnosis is critical to preventing disease progression, as reflected by CD4 counts and viral load (VL). CD4 count measures immune function through the number of CD4 T‐cells per cubic millimeter of blood, while VL indicates the amount of HIV in the bloodstream. HIV progresses through three stages—acute infection, clinical latency, and AIDS [20, 21]. The WHO classifies HIV into four stages, whereas the CDC uses three, combining WHO stages 2 and 3 to align with its ART guidelines. Early ART, especially during Stage 2, preserves immune function and reduces HIV‐related complications. Despite expanded ART access, delays and adherence challenges persist [20, 21].

### 1.4. Barriers to Early ART Initiation

Several psychosocial and structural barriers delay ART initiation. These barriers include fear of a positive HIV test, difficulty accepting an HIV diagnosis [22], and misconceptions about the necessity of ART. Psychosocial characteristics linked to late HIV testing and subsequently late ART initiation include male gender, low education, and overweight status [23, 24]. Studies, such as Su et al.’s research in Yuxi, China [24], reveal that delayed testers often experience slower recovery, higher mortality, and increased loss to follow‐up compared to those who initiate treatment promptly. Prolonged waiting times before treatment significantly heighten these risks.

Clinical and systemic factors delaying ART initiation include high baseline CD4 counts, delays in ART eligibility assessments [25], concerns about side effects, and misinformation about alternative health measures. Research underscores the importance of reducing the time between diagnosis and treatment initiation [26, 27] to aid psychological acceptance and enrollment in lifelong therapy. Delayed ART initiation [28] exacerbates comorbidities like tuberculosis [29] and Hepatitis C [22], further complicating disease management.

### 1.5. The Need for Interventions

To combat HIV progression and its consequences, early and sustained ART adherence is vital for extending life expectancy, preventing HIV transmission, and improving public health outcomes. Community‐based testing, patient‐centered counseling, and removal of eligibility bottlenecks [30] are essential to overcoming barriers and ensuring timely initiation and adherence to ARV treatment for individuals across their life course.

### 1.6. Purpose of Study

This study investigates the interval between HIV diagnosis and ART initiation, as well as the barriers to ART adherence among men living with HIV in Greater Gaborone, Botswana. Women are included as a comparison group to contextualize male‐specific challenges [31, 32].

A separate study in Gaborone reported that men were three times more likely to be nonadherent to ART (aOR 3.29, 95% CI 1.13–9.54 [33]). This occurs despite men having a lower HIV prevalence compared to women prevalence in the study area and the country as a whole (14.2% in 2008, 14.1% in 2013%, and 15.2% in 2021) compared to women (20.4% in 2008, 19.2% in 2013%, and 26.2% in 2021) [34]. While men have a lower HIV prevalence, their ongoing challenges with ART adherence compromise the effectiveness of treatment and prevention efforts. Poor ART adherence not only heightens the risk of HIV transmission but also hinders progress in reducing the overall burden of the epidemic. Botswana is one of four countries in the Southern Africa region with the highest HIV prevalence among adults in the world—Eswatini (27.3 per 100 population), Lesotho (23.6), Botswana (20.3), and South Africa (20.4) [9]. Of these, South Africa has the greatest number of people living with HIV (PLH) (*n* = 7.5 million, total population of 60 million people) followed by Botswana (*n* = 350,000, total population of 2.4 million people) [9]. Botswana was the first African country to provide its citizens with free ART in 2002 yet remains a country most affected by HIV, and is therefore the focus of this study [32].

## 2. Materials and Methods

### 2.1. Study Area

This study was conducted in the Greater Gaborone area of Botswana, which comprises the capital city of Gaborone city and three surrounding districts: South East, Kgatleng, and Kweneng East. These areas include a mix of urban, peri‐urban, and rural settings. According to the last national census in 2021, the city of Gaborone had a population of *n* = 246,325, while the larger metropolitan area had 421,907 people [31, 35]. There were 311,026 PLH and on ART in 2021, and from these, 96,331 were residents of the current study area (Table 1). Gaborone had the highest number of people living followed by Kweneng East, Kgatleng, and South East [36].

**Table 1:** People living with HIV (PLH) and on antiretroviral therapy (ART)^a^ in Greater Gaborone, Botswana, 2021.

Area	HIV prevalence *N* (%)
Gaborone	57,224 (11.1)
South East	5245 (12.8)
Kgatleng	13,776 (19.3)
Kweneng East	20,086 (18.7)
Total PLH and on ART	96,331^b^ (30.9)

^a^Source: Botswana Ministry of Health (MoH), 2021.

^b^Out of 311,026.

Although ART uptake is not the focus of this study, understanding ART adherence in high HIV prevalence context remains critical. Adherence rates have been found to be 95% [37] and 99% [33] (with males having a suboptimal adherence of 65%) in studies conducted in Gaborone. ART adherence was found to be 86% among rural PLH in Botswana.

### 2.2. Study Participants

Structured interviews using mixed format questionnaires were administered to 667 adults including 239 men and 428 women living with HIV and attending 21 HIV treatment clinics in the Greater Gaborone area. The questionnaire design consisted of closed‐ and open‐ended questions, reflecting on retrospective recall of events, as well as current knowledge, attitudes and beliefs, and behavioral practices. Participants attended the HIV clinic monthly, where they received ARV medications after a doctor’s consultation. The date when one was first diagnosed with HIV,date of ART initiation, height, weight and biological data such as CD4 count and VL were colleted from the patients’ medical cards. Patients were required to bring their medical cards that the physician would update following their visit. While patients generally had three to four blood tests per year to evaluate their CD4 and VL levels, only the most recent laboratory test was recorded for this survey. Further details on the study population have been described elsewhere [31, 32].

### 2.3. Sample Size Calculation

#### 2.3.1. District Selection (*n* = 4)

The selected study area has a high HIV prevalence, and high number of PLH found to be 96,331 (30.9% of the national total of 311,026) [34] as well as a high number of HIV treatment clinics (100 out of a total of 526 public clinics, 19%) [34] attending to patients receiving ART. Purposive sampling, a nonprobability sampling method, was used in this study. In this approach, “the principal researcher deliberately selected specific elements or subjects for inclusion in the study in order to ensure that the elements would have certain characteristics relevant to the study [38]”. Then, the study area encompassed Gaborone (urban), South East (peri‐urban), and the Kgatleng and Kweneng East districts (both rural). These areas were selected based on their geographic diversity and HIV prevalence. While nonprobability sampling method ensured geographic diversity, it limits the extent to which the findings can be generalized, as noted in the Study Limitations and Strengths section.

#### 2.3.2. Clinics Selection (*n* = 21)

Clinic sampling was guided by the national HIV prevalence (20.3%) since the study did not consider districts as rigid boundaries. There were 100 HIV treatment clinics in the study area, 25 in Gaborone (11.1% HIV prevalence), 6 in South East (12.8%), 26 in Kgatleng (19.3), and 33 in Kweneng East (18.7) [34]. Using the national prevalence (0.20 ∗ 100 clinics), 20 clinics were recommended. To account for over‐ and undersampling of facilities, the number was increased to 21 clinics [31, 32]. The clinics were then purposively selected based on geographic accessibility and logistical feasibility. While this method ensured geographic and clinic‐type diversity, it limits the generalizability of the findings, as discussed in the Study Limitations and Strengths section. Out of the 25 clinics in Gaborone, 5 were selected for this study, which included busy (Old Naledi, Phase 2, Bontleng) and less busy clinics (Broadhurst Traditional and Village (Ext 15) clinics). Clinics were also stratified by income (low‐, middle‐, and high‐income neighborhoods). Four out of six clinics in South East district were selected, while 9 out 33 in Kweneng East and 3 out of the 26 clinics in Kgatleng districts. The same procedures applied in Gaborone were used in the peri‐urban and rural districts with an exception of income stratums.

#### 2.3.3. Sample Size Determination

The inclusion criteria were age 18 years and older women and men who were living with HIV, attending the clinic for ART. Pregnant women or mothers taking ART for prevention of mother‐to‐child transmission (PMTCT) were excluded from this study due to the unique challenge they may experience adhering to ART. Pregnant women receive special care, visiting clinics more than once a month, which may confound comparisons with nonpregnant women. The second stage involved the selection of adult women and men attending clinics in the chosen districts.

#### 2.3.4. Sampling Procedures

The sample size for the participants was calculated using the equation below: Equation (1) was adopted from a national stigma associated with HIV and AIDS survey conducted in 2014 [31, 32, 39].

(1) N=z2 p1−pd2,

*N* = minimum sample size. *z* = level of confidence according to the standard normal distribution (level of confidence of 95%, *z* = 1.96). *p* = estimated prevalence (20.3%) in targeted population (national adult HIV prevalence). *d* = tolerated margin of error (5%)

**Figure 1:**
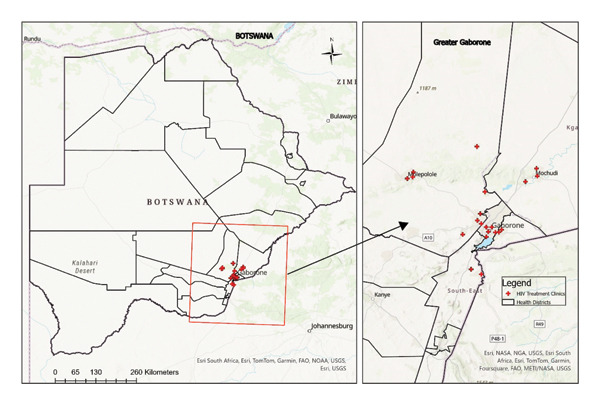
Study areas showing HIV treatment clinics attended by participants in Greater Gaborone, Botswana.

Formula:

(2) N=1.962∗0.203∗10.203−0.052=246,

The minimum sample size needed for the study was 246 adults living with HIV and receiving ART. To ensure stronger and more reliable results, this number was increased to 667. The increase was based on the availability of more patients at each clinic after reaching the initial target of 30 participants per clinic. More than 30 participants were enrolled at clinics that were considered busy, such as Nkoyaphiri clinic in Kweneng East, Old Naledi clinic in Gaborone, and Mafitlhakgosi clinic in South East districts. To get the number of patients per clinic, the power to reject the null hypothesis was assessed in Open Epi Software [40]. The power of a statistical test is the probability that the test will correctly identify Type II error [41]. Failing to reject a false null hypothesis is called a Type II error [41]. To reduce the likelihood of this error, a study needs higher statistical power, which typically requires a larger sample size [42]. The power was set at 80%, meaning that the study will be able to detect a true effect of the ART nonadherence 80% of the time assuming that Type II error would occur only 20% of the time. Open Epi results showed that the required sample size to detect a 20% difference in ART adherence with 80% power, a one‐tailed test [43] at *α* = 0.05 (the outcome variable will be in one direction: ART nonadherence), and an HIV prevalence of 20.3% were approximately 10 participants at each HIV treatment clinic [40]. To detect a true effect of the ART nonadherence 80% of the time assuming Type II error of 20% of the time, the study targeted *n* = 30 participants per clinic, which will total 630 (21 clinics ∗ 30), and the final sample was *n* = 667 (94% response rate), which was a large sample size to detect the expected difference. This larger‐than‐required sample ensured that the study maintained adequate power, providing confidence that any true effect could be detected and that the null hypothesis could be reliably rejected if warranted. Participants came from four districts (*n* = 4) that included urban, peri‐urban, and rural areas. All 21 HIV treatment clinics in these districts were included in the study [32]. The clinics were geocoded in Google Earth to produce a KMZ file, which was later converted to a geographic shape file to show clinics that the participants attended in the study area (Figure 1) in ArcGIS 10.6 [44].

### 2.4. Ethical Considerations

Given the sensitivity of this research, an approval from the Institutional Review Board (IRB) # x15‐1269e was secured from both Michigan State University and the Ministry of Health (MoH) in Botswana prior to administering the survey [32]. After satisfying the requirements of the MoH IRB requirements, to obtain access to the different clinics, separate IRBs were sought from the District Health Management Teams (DHMTs) in Botswana.

Following the selection of clinics and participants, systematic random sampling was employed to select the participants at their respective clinics. Every second participant to enter the door was selected until the minimum required sample of *n* = 30 participants per clinic was reached. This approach avoided exclusion bias of individuals or cultural groups. Informed consent was obtained by the researchers by reading a prepared statement concerning the purpose of the study that participation was voluntary and the participant could withdraw from the interview at any time. The verbal consent by the participant was obtained following this preamble. In addition, they signed the written consent to affirm their agreement to participate in the study and obtain access to their medical records. The interviews took place in an interview room identified by the health personnel [31, 32].

### 2.5. Questionnaire Content Development and Validation

The mixed‐format questionnaires were administered face to face in Setswana (national language) and English (official language). For reliability purposes, all the patients were visited at their respective clinics at the same time (from 7:30 am until 2:00 pm). Four experienced research assistants assisted the principal author with the data collection. The data were coded by the first author to minimize bias that could have occurred if multiple assistants had been involved in the coding process. Two questionnaires were piloted in an HIV treatment clinic that was not in the study area to see how long the questions took and to assess the clarity of the questions asked. Below is the description of the questions in the questionnaire as well as measurement of ART adherence.

### 2.6. Data

#### 2.6.1. Definition of ART Adherence

ART adherence in this study was defined by three variables [1]: Biological: patient’s CD4 count ≥ 350 cells/mm^3^ (CD4); [2] Survey: Did you take your pills as instructed by the doctor (100% dosage)? (ART Prescribe); and [3] do you miss a (one) dosage sometimes? (MissDose) (97% or at least missed 1 pill a month). ART adherence was defined as > 97% (missing no more than one dose per month). This threshold was selected to align with Botswana’s national ART program standards [45], which emphasize near‐perfect adherence to maintain viral suppression and minimize resistance. While many studies use a 95% cut‐off [46–49], the > 97% criterion reflects the high adherence expectations in local clinical practice and national treatment guidelines. Participants were categorized as ART adherent (coded = 0) if they had CD4 ≥ 350 cells/mm^3^, reported taking ART as prescribed (100%), and missed no more than one dose per month. Failure to meet one or more criteria resulted in classification as “Non Adherent” (coded = 1). In this study, the dependent variable in subsequent statistical models was “ART Non‐Adherence”. The CD4 count was also evaluated as a continuous variable (range: 50–1450 cells/mm^3^) after preliminary analysis revealed the CD4 count was the primary evidence for ART nonadherence. The most recent CD4 count and VL were obtained from the patient’s medical record with verbal permission from the patient and provided by the healthcare provider. At the time of this study (2016), the Treat All Program was not yet rolled out in Botswana. The CD4 count of < 350 copies/mm^3^ and later on < 500 copies/mm^3^, thresholds were used to enroll those living with HIV into the ART program. VL data were not used to define adherence due to high right‐skewness (range: 24–7506 copies/mL; mean = 472.4; skewness = 8.7; kurtosis = 96.8). Participants with VL > 7000 (*n* = 22) were excluded. Consistent with previous studies in Botswana [50, 51], most participants were on track to meet the UNAIDS 90‐90‐90 targets [50, 51].

#### 2.6.2. Independent Variables

The explanatory (independent) variables to predict ART nonadherence were coded as gender (women = 0, men = 1); age groups—(base, 30–39 years) = 0, and all others are dummy coded as 1, based on previous literature on ART nonadherence [52]. Body mass index (BMI) was calculated from height and weight as an indicator of overall nutritional status. The year of HIV diagnosis (HIV_Year) and the year ART was initiated (ART_Year) were analyzed using both continuous annual data and categorized time periods to study the life course. Life‐course categories were as follows: (1) the first patient reported being diagnosed with HIV in Botswana in 1985 (Category 1: 1982–1985), (2) universal access to free ART was introduced in Botswana in 2002, and the study was conducted in 2016 when the CD4 count threshold was used to determine when ART should begin (Category 2: 2002–2016). Other explanatory variables included any illness/condition (Illness); this variable was coded as 1, and 0 for no illness. To understand the side effects of ART, the question on how they felt after taking ART (ARTFeel), was asked, and if one felt no side effects after a dose, the response was coded as a 0. Additionally, the variable “SexPartner,” which represented the number of sexual partners someone had over the past year, was included in the analysis. Extreme values, such as reports of 16 partners within 12 months (*n* = 2), were removed. This left a total of 658 men and women in the analysis [31, 32]. Finally, open‐ended questions were analyzed using thematic analysis to enhance the results, providing a richness of the discussion points. Analytical models excluded pregnant women or mothers taking PMTCT (*n* = 67) due to unique challenge they may experience adhering to ART.

### 2.7. Descriptive and Inferential Analyses

#### 2.7.1. Quantitative Analysis

Men and women were stratified by ART nonadherence and sociodemographic and facility characteristics to assess their differences using chi‐square tests. The stage of HIV disease (using the CDC stages) at time of HIV diagnosis were calculated for men and women by calculating the number of years between HIV diagnosis and ART initiation (Stage 1, ≤ 1 year; early Stage 2, 2–5 years; late Stage 2, 6–9 years; and Stage 3, 10+ years). The eligibility threshold for ART initiation has changed over time. The baseline CD4 cell count cut‐off increased from ≤ 200 cells/mm^3^ to 201–350 cells/mm^3^ for WHO Stage III (CDC Stage 2), then to ≤ 350 cells/mm^3^, and later to ≤ 500 cells/mm^3^ [20, 21]. In this study, the staging followed these historical thresholds. To reflect the brief transition period between the 350 and 500 cells/mm^3^ cut‐offs in Botswana at the time of data collection, Stage 2 was further divided into early and late Stage 2. The age groups were categorized into 10‐year intervals to balance precision and practicality in analyzing ART adherence behaviors. While narrower age (5‐year intervals) categories could capture more specific differences, they could also result in small sample sizes within each subgroup, leading to statistical limitations and less reliable conclusions.

#### 2.7.2. Logistic Regression

Finally, logistic regression models were estimated to investigate the odds of ART nonadherence for men vs. women at different age groups with men = 1 based on previous literature [31–33]. Unadjusted and adjusted models were estimated using the duration of time from HIV diagnosis to ART initiation. While grouping the data into larger intervals helped to reduce the presence of imbalanced data in preparation for the regression models, some imbalance remained. For example, within the age variable, women aged 30–39 years had the highest frequency (*n* = 148) compared to men in the same age group (*n* = 60). To address this and other imbalanced data, this study estimated logistic regression models using the least squares (LS)‐means procedure (LSMEANS) to estimate the predicted population marginals in SAS 9.4 [53]. The LSMEANS approach was selected because it adjusts for unequal sample sizes across groups and produces predicted population marginals, which are adjusted means accounting for covariates. This method is useful for imbalanced data because it allows fair comparisons by presenting means as if all groups had the same covariate distribution [53]. The linear functions were defined by population marginal means of the corresponding within group means for balanced data. The differences inLS means were then displayed as odds ratios (OR) using the ODDSRATIO option [54]. Further, when performing an LS‐means procedure with logistic regression in SAS, it produced a diffogram, and OR with 95% confidence intervals. In a diffogram, each line segment corresponded to one pairwise difference between LS‐means. A line segment centered at the LS‐means in a pair and had both a vertical and a horizontal line that indicated values and levels that corresponded to the pair. The length of the line segment reflected the projected width of a confidence interval for the difference [54]. In the models (logistic regression), the unadjusted and adjusted models controlled for potential confounders (detailed in the data section). These variables included demographic variables such as sex, lifestyle variables (BMI, HIV status, any illness, sexual partners), and socioeconomic variables (occupation, education). Chi‐square tests were used for preliminary variable selection, with those showing *p* < 0.20 considered for inclusion in the logistic regression model. Multicollinearity was assessed by calculating VIFs for all candidate variables, and those with a VIF greater than 5 were excluded [55]; for example, education (VIF = 5.5) was dropped because it exceeded the acceptable threshold.

#### 2.7.3. Qualitative Analysis

In addition to quantitative data, qualitative data (from the open‐ended questionnaire) was used to complement the findings. The questions included resources at home and at the HIV treatment clinics. Thematic analysis, as outlined by Braun and Clarke (2006) [56], was employed to identify, analyze, organize, describe, and report themes within the data set. Following their six‐step approach, three major themes emerged: economic, environmental, and lifestyle factors influencing ART adherence. Direct quotes were incorporated to emphasize key meanings. By adhering to this structured process, the study ensured logical rigor and falsifiability, enhancing confidence in the generalizability of the results.

## 3. Results

### 3.1. ART Nonadherence for Men and Women

Participants ART nonadherence in Greater Gaborone was 46.0% (adherence 54.0%) for men and 29.1% (adherence 70.9%) for women (Table 2). The overall ART adherence in the study area was 62.5% (ART nonadherence of 37.5%). For men and women 18–29 years, ART nonadherence was slightly lower for men (4.6%) compared to women (6.2%). That gap narrowed for men and women aged 30–39 years (11.7% vs. 11.6%). Thereafter, men were substantially more ART nonadherent compared to women at ages 40–49 years (16.7% vs. 8.6%) and 50+ years (12.9% vs. 2.9%). By marital status, both men and women were mostly either single or cohabiting. Single men tended to be more ART nonadherent (21.3%) than single women (15.9%). There were more men 15.9% (7.9% nonadherent) compared to women 5.5% (1.2% nonadherent) who did not have education. There were also fewer men (5.4%) compared to women (10.2%) with a university education, with both having similar ART nonadherence (about 3% each). These differences in ART nonadherence between men and women by age, marital status, and educational levels were not statistically significant. A majority (41.1%) of women were unemployed compared to men (19.7%). There were significant differences in occupations for men and women and ART nonadherence (*X* ^2^ = 9.96, *p*‐value = 0.04). Of the service‐centered characteristics, clinic utilization showed that a majority of both men (55.6%) and women (57.0%) attended rural clinics. Of these, 24.3% men were ART nonadherent compared to 14.8% of women. ART nonadherence was double for men who lived in peri‐urban areas (13.8%) compared to 6.4% of women in these same areas. Men and women attending urban clinics had the same percentage of ART nonadherence (7.9%). These urban, peri‐urban, and rural differences in ART nonadherence between men and women were also statistically significant (*X* ^2^ = 6.2, *p*‐value = 0.05). Across all sociodemographic characteristics, men showed higher trends of ART nonadherence compared to women.

**Table 2:** Sociodemographic characteristics of sampled women (*n* = 419) and men (*n* = 239) living with HIV in Greater Gaborone, Botswana 2016.

Characteristics	Women		Nonadherence	Men		Nonadherence	Nonadherence vs. adherence	
							Women	Men
	No.	(%)	(%)	No.	(%)	(%)	Chi‐square (*p*‐value)	
Age (years)							2.4 (0.49)	2.16 (0.53)
18–29	91	21.7	6.2	30	12.6	4.6		
30–39	148	35.3	11.5	60	25.1	11.7		
40–49	124	29.6	8.6	90	37.7	16.7		
50+	56	13.4	2.9	59	24.7	12.9		
Marital status							0.85 (0.93)	1.18 (0.75)
Married	60	14.3	4.3	39	16.3	7.5		
Single	223	53.2	15.9	105	43.9	21.3		
Cohabit	118	28.2	7.9	88	36.8	15.5		
Widowed	17	4.1	0.9	7	2.9	1.7		
Education							0.71 (0.94)	7.16 (0.12)
No. Formal	23	5.5	1.2	38	15.9	7.9		
Primary	89	21.2	6.2	73	30.5	14.6		
Secondary	249	59.4	17.4	100	41.8	19.7		
Vocational	16	3.8	1.2	15	6.3	0.8		
University	42	10.2	3.1	13	5.4	2.9		
Occupation							**9.96 (0.04)**	7.16 (0.12)
Unemployed	172	41.1	10.7	47	19.7	8.8		
Self‐employed	70	16.7	4.3	57	23.9	11.3		
Farmer	14	3.3	1.9	14	5.9	2.9		
Private	99	23.6	8.6	82	34.3	15.5		
Government	64	15.3	3.6	39	16.3	7.5		
Clinic Location							**6.2 (0.05)**	1.7 (0.41)
Urban	82	19.6	7.9	44	18.4	7.9		
Peri‐urban	98	23.4	6.4	62	25.9	13.8		
Rural	239	57.0	14.8	133	55.6	24.3		
Total	419	100.0	29.1	239	100.0	46.0		

*Note:* Total: All participants (*n* = 658 with *n* = 419 women and *n* = 239 men). Bolded *p* values are significant at *p* < 0.05.

### 3.2. Stage of HIV Disease at Time of Diagnosis

Men were diagnosed with HIV at different age groups across the life course. Table 3 shows these age groups and stages of HIV disease at time of HIV diagnosis. Most men (69.1%) were diagnosed in Stage 1 of HIV disease compared to early Stage 2 (19.2%), late Stage 2 (7.1%), and Stage 3 (4.6%). Men diagnosed in Stage 1 were 40–49 years (38.2%) and 30–39 years (23.0%). These same age groups had the highest percentage of men diagnosed in early Stage 2 (30.4%, 37%) and 40–49 years had the highest percentage in late Stage 2 (45.5%); however, for ages 30–39 years, the percentage of men diagnosed in early Stage 2 (30.4%) was higher than men diagnosed in Stage 1 (23.0%) in this same age group. There were 11 men (4.6%) diagnosed in Stage 3 with most men 40–49 years (*n* = 5) followed by 30–39 (*n* = 2) and 50–59 (*n* = 2) years. Of those men diagnosed in Stage 3, the delay in ARV initiation from time of HIV diagnosis ranged from 10 years to 19 years.

**Table 3:** Stage of HIV diagnosis for men (*n* = 239) by age group, Greater Gaborone, Botswana, 2016.

	**Stage 1**		**Early Stage 2**		**Late Stage 2**		**Stage 3**		**Total**	
**Age (years)**	***N***	**(%)**	***N***	**(%)**	***N***	**(%)**	***N***	**(%)**	***N***	**(%)**

18–24	9	5.5	0	0.0	2	11.8	1	9.1	12	5.0
25–29	14	8.5	3	6.5	0	0.0	1	9.1	18	7.5
30–39	38	23.0	14	30.4	6	35.3	2	18.2	60	25.1
40–49	63	38.2	17	37.0	5	29.4	5	45.5	90	37.7
50–59	30	18.2	6	13.0	2	11.8	2	18.2	40	16.7
60+	11	6.7	6	13.0	2	11.8	0	0.0	19	7.9

All Ages	165	69.1	46	19.2	17	7.1	11	4.6	**239**	100.0

*Note:* 4 stages of HIV diagnosis include (i) Stage 1, (ii) early Stage 2, (iii) late Stage 2, and (iv) Stage 3. Bold value represents the total number of men (all ages).

Women were also diagnosed with HIV at different age groups across the life course. Table 4 shows these age groups and stages of HIV disease at time of HIV diagnosis. Most women (62.5%) were diagnosed in Stage 1 of HIV disease compared to early Stage 2 (23.2%), late Stage 2 (8.4%), and Stage 3 (6.0). There were higher percentages of women diagnosed in early Stage 2 in 25–29 years compared to Stage 1 (18.6%, 17.9%) and 30–39 years (42.3%, 30.9%). There were 25 women diagnosed in Stage 3 with most women aged 30‐39 years (n=13) and 40–49 years (*n* = 11). Of those women diagnosed in Stage 3, the delay in ARV initiation from time of HIV diagnosis ranged from 10 to 21 years (Table 4).

**Table 4:** Stage of HIV diagnosis for women (*n* = 419) by age group, Greater Gaborone, Botswana, 2016.

Age (years)	Stage 1		Early Stage 2		Late Stage 2		Stage 3		Total	
	*N*	(%)	*N*	(%)	*N*	(%)	*N*	(%)	*N*	(%)
18–24	17	6.5	3	3.1	0	0.0	1	4.0	21	5.0
25–29	47	17.9	18	18.6	5	14.3	0	0.0	70	16.7
30–39	81	30.9	41	42.3	13	37.1	13	52.0	148	35.3
40–49	77	29.4	25	25.8	11	31.4	11	44.0	124	29.6
50–59	30	11.5	7	7.2	4	11.4	0	0.0	41	9.8
60+	10	3.8	3	3.1	2	5.7	0	0.0	15	3.6

All Ages	262	62.5	97	23.2	35	8.4	25	6.0	**419**	100.0

*Note:* 4 stages of HIV diagnosis include (i) Stage 1, (ii) early Stage 2, (iii) late Stage 2, and (iv) Stage 3. Bold values show the total for women (all ages).

### 3.3. Gender‐By‐Age Risks for ART Nonadherence

The unadjusted logistic models (Table 5) showed that men had significantly higher odds of ART nonadherence compared to women at ages 50+ years [OR 4.06, 95% CI 1.79–9.12] and 40–49 years [OR 1.88, 95% CI 1.06–3.56]. When controlling for the number of years ART initiated post‐HIV diagnosis, the odds for men compared to women remained relatively similar at ages 50+ years [OR 3.95, 95% CI 1.73–8.97] and 40–49 years [OR 1.91, 95% CI 1.06–3.45] with 30–39 years now significant [OR 2.05, 95% CI 1.06–3.96].

**Table 5:** Unadjusted and adjusted^1^ odds ratio estimates of antiretroviral (ARV) nonadherence for men (*n* = 239) vs. women^2^ (*n* = 352) living with HIV, Greater Gaborone, Botswana, 2016.

**Sex by age**	**Unadjusted** **odds ratio**	**95% LCI**	**95% UCI**

Men vs. Women 18–29 Years	1.30	0.52	3.23
Men vs. Women 30–39 Years	1.89	0.99	3.58
**Men vs. Women 40–49 Years**	**1.88**	**1.06**	**3.56**
**Men vs. Women 50+ Years**	**4.06**	**1.79**	**9.12**

**Sex by age**	**Adjusted** **odds ratio**	**95% LCI**	**95% UCI**

Men vs. Women 18–29 Years	1.24	0.50	3.11
**Men vs. Women 30–39 Years**	**2.05**	**1.06**	**3.96**
**Men vs. Women 40–49 Years**	**1.91**	**1.06**	**3.45**
**Men vs. Women 50+ Years**	**3.95**	**1.73**	**8.97**

*Note:* 95% lower confidence intervals (LCI) and upper confidence intervals (UCI). Bolded *p* values are significant at *p* < 0.05.

^1^Adjusting for duration of time from HIV diagnosis to ART initiation.

^2^Excludes women on PMTCT (*n* = 67).

Table 6 shows that men aged 50+ compared to women of the same age had the highest odds of being ART nonadherent [OR 3.95, 95% CI 1.74–8.97] controlling for the duration of time between HIV diagnosis and ART initiation. Men aged 50+ years also had a higher odds of ART nonadherence compared to women 40–49 years [2.64, 95% CI 1.37–5.10], women 30–39 years [OR 2.62, 95% CI 1.36–5.05], and women 18–29 years [OR 2.44, 95% CI 1.17–5.09]. Men 40–49 years vs. women aged 50+ years [OR 2.86, 95% CI 1.32–6.16] and men aged 30–39 years vs. women aged 50+ years [OR 3.09, 95% CI 1.36–7.03] also demonstrated a significantly higher odds of ART nonadherence, controlling for the duration of time between HIV diagnosis and ART initiation.

**Table 6:** Adjusted^1^ odds ratio estimates of antiretroviral (ARV) nonadherence for men (*n* = 239) vs. women^2^ (*n* = 352) by age group.

Gender	Age	*N*	Gender	Age	*N*	Odds ratio	95% LCI	95% UCI
Men	18–29	30	Women	18–29	91	1.24	0.50	3.11
Men	18–29	30	Women	30–39	148	1.33	0.57	3.14
Men	18–29	30	Women	40–49	124	1.35	0.57	3.16
Men	18–29	30	Women	50+	56	2.01	0.75	5.41
Men	30–39	60	Women	18–29	91	1.91	0.91	3.99
**Men**	**30–39**	**60**	**Women**	**30–39**	**148**	**2.05**	**1.06**	**3.96**
**Men**	**30–39**	**60**	**Women**	**40–49**	**124**	**2.07**	**1.07**	**3.99**
**Men**	**30–39**	**60**	**Women**	**50+**	**56**	**3.09**	**1.36**	**7.03**
Men	40–49	90	Women	18–29	91	1.76	0.89	3.47
**Men**	**40–49**	**90**	**Women**	**30–39**	**148**	**1.89**	**1.05**	**3.41**
**Men**	**40–49**	**90**	**Women**	**40–49**	**124**	**1.91**	**1.06**	**3.45**
**Men**	**40–49**	**90**	**Women**	**50+**	**56**	**2.86**	**1.32**	**6.16**
**Men**	**50+**	**59**	**Women**	**18–29**	**91**	**2.44**	**1.17**	**5.09**
**Men**	**50+**	**59**	**Women**	**30–39**	**148**	**2.62**	**1.36**	**5.05**
**Men**	**50+**	**59**	**Women**	**40–49**	**124**	**2.64**	**1.37**	**5.10**
**Men**	**50+**	**59**	**Women**	**50+**	**56**	**3.95**	**1.74**	**8.97**

*Note:* 95% lower confidence intervals (LCI) and upper confidence intervals (UCI). Bolded *p* values are significant at *p* < 0.05.

^1^Controlling for men vs. men and women vs. women by age group and the number of years ART initiated post‐HIV diagnosis.

^2^Excludes women on PMTCT (*n* = 67).

### 3.4. CD4 Count as a Measure of ART Nonadherence

To further understand gender‐by‐age differences in ART nonadherence, Table 7 shows the most recent CD4 count descriptive statistics for men and women by age group. The mean CD4 count for men was 447.23 cells/mm^3^ compared to 548.93 of the women. Men aged 30–39 years had the lowest mean CD4 count 428.68, followed by men aged 50+ years 429.81 cells/mm^3^. The youngest men (20–29 years) had the highest CD4 count (470.33 cells/mm^3^), which was less than the lowest CD4 count for all women. Women aged 50+ years had the highest mean CD4 count (566.27 cells/mm^3^) followed by women aged 18–29 years with 555.09 cells/mm^3^. The CD4 count for men 50+ years was 136.46 cells/mm^3^ lower than women of the same age. The CD4 mode for men was 200 copies/mL compared to women (391–400 cells/mm^3^) across all ages. While compared to fellow men, it was 300 cells/mm^3^ for men aged 30–39 years, for men aged 50+ years (200 cells/mm^3^) and 400 cells/mm^3^ for men 20–29 years and 40–49 years. These findings indicate that the CD4 count was an important indicator of ART nonadherence among men in this study.

**Table 7:** Descriptive statistics of CD4 count for sample of women^1^ (*n* = 352) and men (*n* = 239) living with HIV, Greater Gaborone, Botswana, 2016.

Sex by age	*N*	Mean	St. dev.
**Women**	**352**	**548.9**	**225.2**
Ages 18–29	65	555.1	249.7
Ages 30–39	117	542.6	232.7
Ages 40–49	114	543.4	208.6
Ages 50+	56	566.3	216.8
**Men**	**239**	**447.2**	**199.5**
Ages 20–29	30	470.3	149.9
Ages 30–39	60	428.7	198.8
Ages 40–49	90	463.3	195.5
Ages 50+	59	429.8	227.8

*Note:* Bolded here differentiate men from women.

^1^Excludes women on PMTCT (*n* = 67).

Table 8 shows that the CD4 intercept for men aged 30–39 years was 584.19 cells/mm^3^ controlling for significant risk factors, including “was sick during the last 2 months” (*β* = −99.39, *p* = 0.0092) and “taking other drugs” (*β* = −98.74, *p* = 0.04). Therefore, the low CD4 count of men of this age group is largely explained by having comorbidities and taking other drugs possibly for those conditions. The CD4 intercept for men aged 40–49 years was 547.14 cells/mm^3^ controlling for risk factors of which VL (*β* = −0.11, *p* = 0.0032) was significant. Finally, the intercept for men aged 50+ years was 833.71 copies/mL, which was largely explained by their single or cohabiting marital status (*β* = −303.21, *p*‐value = 0.0009).

**Table 8:** Regression adjusted estimates of CD4 count for men by age group (*n* = 239) living with HIV, Greater Gaborone, Botswana, 2016.

	***β***	**SE**	***t*-value**	**Pr > (t)**

Men 30–39 years (*n* = 60)				
Intercept	584.19	35.35	16.52	< 0.0001
**Taking other drugs**	**−98.74**	**47.74**	**−2.07**	**0.0404**
**Sick in last 2 months**	**−99.39**	**37.65**	**−2.64**	**0.0092**
Mean BMI	9.28	2.76	3.35	0.0010
ARVs initiated < 2012	89.33	36.24	2.46	0.0149
Viral load	−0.03	0.04	−0.73	0.4639
Men 40–49 years (*n* = 124)				
Intercept	547.14	35.51	15.41	< 0.0001
Mean BMI	4.71	2.57	1.83	0.0698
ARVs initiated < 2012	85.53	37.51	2.28	0.0244
**Viral load**	**−0.11**	**0.04**	**−3.00**	**0.0032**
Men 50+ years (*n* = 55)				
Intercept	833.71	76.39	10.91	< 0.0001
**Single vs. Cohabiting**	**−303.21**	**84.34**	**−3.60**	**0.0009**

*Note:* Bolded here were variables that were negatively associated with CD4 count for different age groups of men.

### 3.5. Qualitative Results

To complement the quantitative findings, participants were asked open‐ended questions about the barriers they faced in accessing ART. Four key questions were designed to identify relevant themes on economic, environmental, and lifestyle factors influencing nonadherence. The first question asked about whether water shortages at their respective HIV treatment clinics had impacted receiving of clinical services. The second explored whether they had backup water supplies at home and the third how they coped with hot weather. The last question examined the availability of financial and other forms of support. Direct quotes were included to highlight significant insights shared by the participants.

#### 3.5.1. Economic Factors

When a young woman who has stated that she was unemployed was asked her source of financial support to travel to her monthly clinic appointments, she said:

*The reason why the doctors say I am a defaulter, is because I can’t afford to come every month here, I don’t have combi fees (combi is a local term for taxi). Sometimes we are required to come twice a month particularly when the doctor is not available. I don’t have a job, so its expensive to make return trips to the clinics*. (32‐year‐old woman, peri‐urban clinic).

Another participant also revealed that transport costs were determent to attending monthly clinic appointments.

*To come for my refill […pills], I have to borrow money from neighbours. If they don’t have, sometimes, I miss my appointment.* (42‐year‐old man, rural clinic).

*I sometimes miss my dosage because I don’t have food in my house. These ARVs are very strong, you cannot take them on an empty stomach. I am unemployed, my unreliable income comes from piece [part time] jobs, said another participant.* (38‐year‐old man, urban clinic).

#### 3.5.2. Environmental Factors

The questions posed to the participants revolved around the water situation (which was a drought year when the study was conducted) and the hot weather.

*The water people (Water Utilities Corporation) sometimes don’t alert us when they are going to close our taps, so we will not have time to store water in buckets. This is not good because if you are unprepared like that, you can’t cook and bath.* (27‐year‐old woman, peri‐urban clinic).

*Water is most of the time not coming out of the taps in the morning when we have to prepare to the clinic. And it [water] will come late in the afternoon, by that time, the clinic is closed and I can’t go for my appointment.* (51‐year‐old man, peri‐urban clinic).

*In the afternoon, it can be too hot to walk to the clinic, so if you don’t have transport money, it becomes a problem. I usually save transport fare, but when I get to the clinic, we also wait a long time, in the heat. It’s a problem.* (39‐year‐old woman, rural clinic).

#### 3.5.3. Lifestyle Factors

The question that elucidated the lifestyle factors was when participants were asked if they had any questions with regard to the survey. The responses ranged from sources of social/partner support, alcohol intake, and work trips.

*I am an old man and I don’t have a partner. So I am not motivated to attend my clinic appointments because no one reminds me to. I need to get married, but who will I marry when I don’t have money.* (59‐year‐old man, rural clinic).

*I don’t have anyone to take care of me and I live alone. I have multiple diseases that I live with. I have TB, I have liver problems and BP. All these conditions, I have medication for. For TB, in the first month of starting the treatment, I had to temporarily stop the ART because it was too many pills.* (55‐year‐old man, peri‐urban clinic).

*During the weekends, I socialize and drink [alcohol]. It becomes hard to remember to take my medication when I am intoxicated.* (45‐year‐old man, urban clinic).

*I travel a lot for work, it can go for weeks, sometimes months. In between, I don’t usually get time to go for my ART services. But what can I do, I need the job.* (47‐year‐old man, peri‐urban clinic).

*I drink alcohol, a lot of it during pay day (month end). Its during this time, I really default on taking my tablets. Its not like I forget to take them, it just doesn’t make sense to take them with alcohol.* (36‐year‐old man, urban clinic).

Qualitative findings were used to triangulate and reinforce the quantitative results. Four key questions were designed to identify themes on economic, environmental, and lifestyle factors influencing nonadherence. These themes clarified specific quantitative patterns, showing, for example, how economic constraints, such as the cost of transportation to clinics or limited financial resources, can hinder consistent access to medication. Environmental factors, including water shortages and extreme weather conditions further complicate adherence efforts. Additionally, lifestyle challenges, such as work schedules, alcohol consumption, and caregiving responsibilities, may also impact their ability to follow prescribed treatment regimens.

## 4. Discussion

### 4.1. ART Nonadherence Among Women

Women living with HIV in Botswana often face multiple structural and social barriers that contribute to low ART adherence. Factors such as unemployment, differences in rural versus urban healthcare access, HIV diagnosis during reproductive ages with late linkage to care, and limited availability of supportive social services, have been shown to significantly affect their ability to consistently follow treatment.

#### 4.1.1. Unemployment

Occupation and clinic attendance in urban, peri‐urban, and rural areas explained some differences in ART nonadherence among women, which was supported by previous studies [57–60]. In this study, a majority of women were unemployed (41.1%), which was substantially higher than the national average (19.1%) [35]. About one‐quarter of unemployed women were ART nonadherent. Women also expressed their concern that travel to the clinic is expensive and sometimes they need to borrow money to attend their appointment. This implies that unemployment may be a significant barrier to ART adherence among women, potentially due to financial instability, lack of transportation fees, or competing priorities such as childcare. Economic hardship can limit access to healthcare services, highlighting the need for targeted interventions that address financial and social support to improve adherence rates.

#### 4.1.2. Rural/Urban Differences

Differences in clinic accessibility across urban, peri‐urban, and rural areas was another factor that helped explain variations in ART nonadherence among women. In this study, ART nonadherence was high for both genders living and attending clinics in rural areas (women, 14.8% and men, 24.3%). In Kenya, van der Kop et al. [57] found that variations in ART initiation by clinic type are due to differences in healthcare providers’ encouragement of early testing and follow‐up linkage to care. In contrast to the current study, residence and occupation were not statistically significant for ART nonadherence in northern Ethiopia [58], while ART nonadherence was greater in urban areas in Tanzania [59] and the United States [60]. In the Tanzania study, urban areas had more younger males [59]. In the U.S. study, the higher adherence observed among rural veterans was partially, but not entirely, attributed to differences in demographics and coexisting conditions [60]. Rural veterans tended to be older and predominantly white—both factors linked to better ARV adherence [60]. Additionally, they had lower rates of alcohol and substance use disorders, which are commonly associated with poorer adherence [60].

#### 4.1.3. Age at HIV Diagnosis

Age at HIV diagnosis and the stage of disease at treatment initiation was another factor that was important on ART outcomes among women living with HIV in Botswana. A majority of women and men were diagnosed with HIV during their reproductive years. Women, in general, were diagnosed with HIV at a younger age than men. A higher percentage of women initiated ART during late Stage 2 of disease compared to men, who were more likely to begin therapy during early Stage 2 or Stage 3. Adjusting for time from HIV diagnosis to ART initiation, men 30–39 years and older were more likely than women to be ART nonadherent. These findings suggest that although women, in general, initiated ART later in the progression of HIV disease, they were less likely to be ART nonadherent. This could be explained by the fact that women experienced higher CD4 counts (Table 7) compared to men at all ages. In Botswana, women’s lower likelihood of ART nonadherence despite initiating treatment later in HIV progression may be attributed to greater healthcare engagement, stronger social support networks, and adherence‐focused interventions targeting maternal health. A study in Shiraz, Iran, showed that even though women referred later to health facilities (in the late AIDS stage), they visited more regularly and complied better with medication in comparison to men [61]. In Ethiopia, Teklu et al. [27], in a study among *n* = 4159 participants, found that being male and having a low CD4 count were risk factors for HIV‐related mortality. In Cape Town, South Africa, George et al. [62] found that a significantly greater proportion of men (46%) had a low CD4 count (< 350 copies/mm^3^) compared to women (25% *p* ≤ 0.001). In two Tanzanian studies, Muya et al. [63] found that increased CD4 count and duration on ART were associated with ART nonadherence and Semvua et al. [59] also found that patients with higher CD4 counts were at increased risk of ART nonadherence. These findings could be explained by “pill fatigue”, that is, patients who see health improvements by adhering to ART over time begin to relax their practice of taking ART. The disparities in CD4 count and ART duration warrants further exploration.

#### 4.1.4. Availability of Social Services

Accessible and targeted social services play a crucial role in linking women to HIV care and supporting sustained ART adherence. Social services available in Botswana for women include the provision of psychosocial services for young women, female economic empowerment, effective HIV prevention programs, pre‐exposure prophylaxis (PrEP), and PMTCT. Services such as HIV prevention programs are usually targeted to secondary school females which mean those that are out school may be excluded. PrEP is a course of drugs taken by someone who is HIV negative before potential exposure to HIV, to prevent infection [64, 65]. It is available to key groups which include young/adolescent women, sero‐different couples, and female sex workers. Challenges with PrEP include limited availability particularly in remote areas and lack of HIV status disclosure of male partners. To further improve early ART initiation and adherence among women, it is important to encourage and support women to bring in their partners at health facilities. Additionally, it is important to recognize the need for access to clean and safe water and to reduce the burden of travel during very hot days for women traveling to their ART appointments as shown in this study.

### 4.2. ART Nonadherence Among Men

#### 4.2.1. Comorbidities and Concurrent Medication Use

Comorbidities and concurrent medication use were significant factors contributing to ART nonadherence among men aged 30–39 years. Men aged 30–39 years in this study showed that recently being sick and/or having comorbidities and taking other drugs for these illnesses significantly increased ART nonadherence as evidenced by decreased CD4 count. Several authors corroborated this study’s finding that taking medication every day is a challenge for patients with other chronic illness, such as diabetes and hypertension, taking their ART about 70% of the time which could explain nonadherence of men with comorbidities [66–69]. HIV/TB coinfected individuals often require concomitant ART and anti‐TB therapy, which improves survival but presents challenges like drug–drug interactions and overlapping toxicity [68]. Rifampicin, a key TB drug, reduces ART effectiveness by inducing hepatic enzymes, necessitating dose adjustments [69]. Careful monitoring, alternative regimens, and integrated management strategies are essential for optimizing treatment outcomes. In this study, a man in his late 30s was concerned about not having enough food and did not want to take his medication on an empty stomach. Such discussions could be held in the clinic to address men’s concerns and their access to food sources.

#### 4.2.2. Age and Comorbidities

Unlike women, men face age‐specific barriers to ART adherence, including comorbidities, resulting in high VLs and low CD4 counts, as well as limited social support.

Males aged 40–49 years with a high VL were also significantly more likely to be ART nonadherent, also evidenced by a lower CD4 count. In contrast, George et al. [62] in South Africa found that females, patients older than 35 years, smoking, and low CD4 count were associated with detectable high VL. There are social services available for men in Botswana such as voluntary medical male circumcision (VMMC), TB/HIV testing targeting males, and rapid ART initiation as of 2019, which can include same‐day and fast track initiation to ART [70]. Although these are commendable services, some are not age‐specific, which can limit interest. For instance, VMMC is promoted for males aged 15–49 who are HIV‐negative as a preventative HIV measure. Other concerns for men in the 40s were unemployment, the need to borrow money to purchase ARVs, alcohol use, and comorbidities as further described below.

TB and HIV testing services are offered at different facilities which can be inconvenient for men, particularly if they must travel long distances to these health facilities [71]. Rapid ART initiation is only done at government clinics and on business/working days, which means that a majority of working men who are free on weekends, will not have access to this service. Social services recommended for men with HIV and comorbidities include HIV counseling that emphasizes lifestyle modification and self‐management and access to clinics after work hours so that men can access these needed services.

#### 4.2.3. Age and Limited Social Support

Finally, men ≥ 50 years were at increased odds of ART nonadherence if they were single vs. cohabiting with a spouse or partner. Yang et al. [72] found that older men had low levels of social support, which led to ART nonadherence. Abara et al. [73] found that in the United States, older females (50–64 years) with comorbidities were less adherent to ART, based on a study that exclusively interviewed a cohort of patients within this age group. In western Saudi Arabia, Farahat et al. [74] found that HIV patients older than 50 years were at higher odds of having comorbidities and the management of their medications required special care and adequate resources at primary‐and tertiary‐care levels. Nyirenda et al. found in South Africa that among those HIV patients aged ≥ 50 years, levels of happiness and life satisfaction were lower compared to other age groups. Still in South Africa, Nyirenda et al. found that more than 11% of men aged 50–59 had a female sexual partner in the past month aged 31–40 years, demonstrating that intergenerational relationships were sources of HIV transmission [75]. In Botswana, single men who are older may seek younger women as companions, which may contribute to HIV transmission [76]. Future research should prioritize the needs of middle‐aged and older men, as studies such as one conducted in Toronto, Canada [77], have shown elevated suicide rates in these age groups linked to socioeconomic factors, including family breakdown and unemployment. Addressing these issues can inform targeted public health, healthcare, and social service interventions for these vulnerable populations. There is already collaboration between community leaders and the government to promote HIV testing in communities in Botswana [78]. There should, however, be increased engagement of local and faith leaders to promote not only HIV testing but also social support for aging men that includes encouraging them to take their ART medications. This engagement could also address the loneliness experienced by older men.

### 4.3. Study Limitations and Strengths

In this study, (a) survey response bias could be a potential limitation if the participants provided answers they thought the principal researchers wanted to hear. There could also be issues of recall bias as well as under‐ or overestimating ART adherence levels because participants were asked to self‐report when and how they took their medication. Future interventions should consider supplementary measures such as pharmacy refill data and pill count. (b) The study interviewed only patients on ART and did not include patients lost to follow‐up/discontinued ART. These patients, from discussions with health personnel, were not traceable from their cellphone contacts, making it difficult to call them for missed appointments. Patients lost to follow up possibly underestimated ART nonadherence levels in this study’s population. (c) Circular migration from Gaborone to cities and towns in other peri‐urban and rural area districts may influence the clinic a participant attended, which may not be representative of where they lived, making this study area less representative of Botswana as a whole. Our study also had limitations due to its (d) cross‐sectional design, which precludes the establishment of causal relationships between explanatory variables and outcomes. For instance, we cannot infer that comorbidities that the 30–39‐year‐old men experience led to nonadherence or vice versa.

Additionally, the data collected reflects the participants’ current status and does not account for time‐varying factors, such as changes or evolving treatment guidelines. For instance, the study was conducted when the CD4 count threshold was still in place, but this was later replaced by the Treat‐All program, which no longer uses CD4 counts as an enrollment criterion. Another limitation was that (e) purposive sampling was used to select the study area focusing on specific characteristics, but this limits generalizability and introduces potential bias due to the lack of random selection. While participants within the area were randomly chosen to reduce bias and improve diversity, the initial purposive sampling remains a limitation. This needs to be considered when interpreting the results, as it may affect the broader applicability of the findings. Furthermore, findings from Greater Gaborone, Botswana, may not be applicable to other regions or countries with differing healthcare systems, social norms, or economic conditions, which could influence ART adherence. These factors limit the external validity of the results, and thus, the findings should be interpreted with caution. Nevertheless, this study utilized a sensitive measure of ART nonadherence that captured behavioral and biological confirmation among adult men and women regardless of residence and/or place of clinic attendance.

## 5. Conclusions

This study revealed two key findings: women are more likely to adhere to ART but need encouragement to start treatment sooner after diagnosis, while men tend to start ART earlier but are more likely to be nonadherent. Despite free ART and medical care in Botswana, challenges remain, particularly for men who may face barriers related to comorbidities and social support. The study underscores the importance of gender‐specific interventions to improve ART adherence. Education on the significance of timely medication intake, particularly for men, is crucial for viral suppression, disease management, and preventing new infections. Both genders are often diagnosed in their reproductive years, highlighting the importance of successful ART implementation to meet UNAIDS’ 2030 goals. Enhanced awareness and understanding, particularly among older men, are essential for achieving the Treat‐All program’s objectives in Botswana.

## Conflicts of Interest

The authors declare no conflicts of interest.

## Funding

The data collection phase was supported by funds from the Center for Gender in Global Context at Michigan State University.

## Data Availability

Raw data generated in this research and derived data (deidentified data as well as the SAS scripts…/Life‐course_script.sas) supporting this study’s findings are available from the corresponding author (kelepilem@ub.ac.bw) upon request.
